# The State of Occupational Health and Safety Management Frameworks (OHSMF) and Occupational Injuries and Accidents in the Ghanaian Oil and Gas Industry: Assessing the Mediating Role of Safety Knowledge

**DOI:** 10.1155/2020/6354895

**Published:** 2020-03-13

**Authors:** Suxia Liu, Edmund Nana Kwame Nkrumah, Linda Serwah Akoto, Emmanuel Gyabeng, Erasmus Nkrumah

**Affiliations:** ^1^School of Management, Jiangsu University, Zhenjiang, China; ^2^Kwame Nkrumah University of Science and Technology, Kumasi, Ghana

## Abstract

*Background*. The study examines the mediation effect of safety knowledge in causal the relationship between Occupational Health and Safety Management Frameworks (OHSMF) and occupational injuries and workplace accidents in the Ghanaian Oil and Gas Industry. The study explores different dimensions of occupational health and safety management systems, workplace accidents, and occupational injuries. The study adopted a cross-sectional survey design. A total of 699 respondents through a convenience and purposive sampling technique were selected in three government-owned oil and gas organizations for the study. Correlation, multiple regression analysis, and bootstrapping methods were used for data analysis. The findings of both the regression and correlation analysis indicated that there is a moderately strong negative and significant relationship between Occupational Health and Safety Management Frameworks (OHSMF) and workplace accidents and occupational injuries. Safety knowledge significantly mediates the causal relationship between OHSMF and workplace accidents and injuries. Safety training was found to be a significant predictor of safety knowledge, work-related injuries, and workplace accidents. The negative relationship between OHSMF and workplace accidents and injuries shows that the existing OHSMF are either ineffective or lack the acceptable safety standards to control hazard exposures in the industry. Management must invest in frequent safety training and orientations to improve safety knowledge among workers. The study further recommends government and industry players to extend serious attention towards the promotion and improvement of occupational health and safety management systems in Ghana.

## 1. Introduction

The genesis of most disastrous accidents among global industries, in both the present and the past can be traced back to the absence or weak implementation of occupational health and safety management systems. Despite the familiarity of the occupational health and safety concepts, organizations across different sectors continue to record huge losses and inefficiencies due to the high rate of job-related illness and injuries. A report by the International Labour Organization (ILO) on “employees' health and safety” in 2015 convinces that industries need to invest more efforts towards the improvements of employees' health and safety at work. According to the report, over 2.3 million occupational accidents occurred annually around the world, and out of this number, an estimated death of over 6000 employees was recorded daily [1]. However, the rate of work-related injuries and accidents varies across countries depending on the level of industrialization. Specifically, developing countries continue to record huge losses in work-related accidents than the developed countries [2–5].

Ghana falls within these developing countries with the highest estimated accident rate at 15,702 per 100,000 workers and fatal accidents at 1852 with a fatality rate of 20.6 per 100.00 workers across industries [6]. Data from the Department of Factories Inspectorate (DFI) as well suggest that occupational accidents and work-related injuries for the period 2000–2008 cost Ghanaian industries an estimated amount of GHC 2,719,651.92 ($530,000). Thus, averagely, accidents and injuries cost employers approximately GHC 543,930.38 ($60,000) each year and GHC 302.96 ($60) per case, yet, safety issues do not seem to be one of the top most priorities due to several reasons such as the lack of comprehensive health and safety policies, poor infrastructure, and the insufficient number of qualified safety personnel [6, 7].

The oil and gas sector is among one of many industries characterized with convergence of numerous hazardous exposures that can potentially cause serious catastrophes and work-related accidents. Serious catastrophes ever recorded in the oil and gas sector that has claimed life and caused damages to properties include the Piper Alpha disaster in 1988 [8], Montara blowout in 2010 [9], and the Texas City refinery explosion in 2005 [10]. Currently, the oil and gas sector in Ghana remains one of the most productive sectors and obviously falls within one of the riskiest industries in the world. According to Sutton, [11], incidents such as hydrocarbon leakages, falling objects, fires, explosions, blowouts, and hydrogen sulphide emissions are likely exposures to workers in the sector. Thus, from the first phase of the operational activities, consideration must be given to accidents prevention.

In Ghana, a report by the General Reinsurance Africa Ltd. [12] indicated that most serious and dangerous accidents faced by the oil and gas industry are explosions, which mostly kill workers and destroy equipment within the blast area. Other degrees of injuries exposed to employees in this sector as published by the Ghana Health Service and Ministry of Health [13] report include slips and falls, electrical shocks, and burns. A review by Oppong [6] as well revealed that employees in the Ghanaian oil and gas sector suffer from occupational injuries such as contusions, cuts, and laceration of the legs, hand, fingers, and eye. In the existence of all these catastrophic events, poor safety infrastructure, lack of funding for safety systems, the large number of unqualified occupational health professionals, inadequate OHS work-related accident and injury monitoring mechanism, and the unavailability of health and safety data were cited as major challenges industries face in Ghana, including the productive oil and gas sector [7, 14]. Amidst these challenges, the workers remain the victims of most hazards, injuries, and work-related accidents that occur at the workplace.

Hazards and injuries at the workplace affect employees' integrity [15]. Accident causation theories including Petersen's accident-incident theory and the distraction theory have both shown that system failures are mostly the causes of human errors; hence, management holds the obligation to improve job safety while the employees duty is to comply [16]. More importantly, improving safety does not necessarily mean providing safety systems or supportive safety environment for employees alone but as well educating and training workers to improve their safety knowledge. As indicated by Griffin & Neal [17], safety knowledge is a significant determinant of safety performance among employees.

Safety knowledge is basically the degree of employees' knowledge on organizational safety systems, practices, and procedures. According to Campbell et al. [18] and Griffin and Neal [17], individual performance at work is determined by their degree of knowledge, skills, and motivation; hence, employees practice safe behaviour in accordance with safety rules and procedures if they possess these three essentials. Improving employees' safety knowledge also means that organizations must commit enough resources into frequent systematic and comprehensive safety and health training programs for all employees [19]. This was confirmed in the study of Morrison et al. [20] further indicated that organizational climate influences knowledge when employees undergo training, development, and participation in organizational activities. These assertions clearly denote that the level of safety compliance and participation in safety activities is determined by the employees' degree of safety knowledge. Accidents and injuries are expected to become predictable when employees possess the required safety knowledge and undergo frequent safety training. Previous studies suggest that organizations who found it necessary to improve employee safety knowledge through safety training record lower accidents rate [21–23]. In this regard, safety knowledge can be viewed as accident prevention programs which may reduce accident and injury frequency at work.

### 1.1. The Current Study

Accidents of any form may be preventable on a site once good safety planning, management procedures, and cultural practices are rightly placed. Risks can be identified before the onset of any operational activity, and this remains the fundamental principle of accident prevention. Though the causes of accidents and injuries may differ across sectors, the identification and investigation of accidents run on accident causation theories [24]. Heinrich et al. [25] in the late 1920s collected and studied a number of industrial accidents—a total of 75,000 accidents studied revealed that 88% of 75,000 accidents were triggered by risky workers' behaviour. Likewise, the Human Factor Theory of Accident Causation, accident/incident theory, Behaviour-Based Safety (BBS), Turner's model of accident causation, and the Swiss cheese model confirm the assertion of Heinrich [26]. Clearly, the foundation for the majority of accident causation among industries can be attributed to human errors; hence, the burden of accident prevention lies on both management and employees to co-operate at work.

As management strives to provide safety systems to improve job safety, employees must also be prepared to understand these systems and conform to safety rules, compliance and participation. Most of the burden however lies on the organization to support employees through training and education to improve safety knowledge [16, 27, 28]. Safety knowledge has been identified as a significant determinant of achieving the best safety results [17].

Although previous studies have focused on several safety-related issues, including studying the mediating role of safety knowledge in safety management and safety behaviour [17, 28], only few studies have focused on occupational health and safety management systems in the Ghanaian oil and gas sector, with none focusing on work-related accidents and injuries that exist in this highly risky industry [14, 29–31]. This is quite intriguing, as most of the findings established in these previous studies indicated and unraveled numerous lapses in the application of occupational health and safety management systems and policies in this sector. Although the oil and gas sector in Ghana is emerging, there is a vast documentary on serious issues of catastrophes and critical workplace accidents that befalls the global oil and gas sector ([6, 9, 10]); hence, this study questions why an important study such as OHSMF and work-related injuries and accidents have not yet merited any significant research attention in Ghana. Also, studies assessing the mediation effect of safety knowledge between occupational safety systems and work-related accidents and injuries have not also been conducted.

This current study anticipates that, the more reason why occupational accidents and work-related injuries continue to grow in developing countries (e.g., Ghana) cannot only be attributed to the lack of effective implementation of safety management systems but also, the lack of safety knowledge or low safety knowledge among workers; hence, a study of such nature in a risky industry like the oil and gas sector in Ghana is expected to be highly significant to managers, safety practitioners, and policy makers. Again, with regard to the hazardous operational nature of the oil and gas sector in Ghana, safety knowledge is expected to improve safety outcomes. Workers who are likely to engage in unsafe work practices are suspected to be workers who ignore safety rules and procedures due to the ignorance of safety task and procedures. This current study therefore fills this gap by providing an understanding on the mediating effect of safety knowledge in the relationship between OHSMF and workplace accidents and injuries. The findings on the influence of OHSMF on workplace accidents and injuries on each of oil and gas organizations as adopted for the study were also provided in the findings.

## 2. Literature Review

### 2.1. Occupational Injuries, Workplace Accidents, and Safety Management Systems

An injured worker is an inefficient worker; hence, a cost to the organization. Camino [15] posits that occupational accidents can cause a high level of absenteeism among employees and accumulate high cost of production for the organization. Leigh et al. [32] and Steenland et al. [33] posited that each single occurrence of accident causes ten fatal diseases which demands the attention of the organization. Accidents cause discomfort to both the worker and the organization. At worst, accidents that lead to permanent disability may increase cost and slow production as companies take responsibilities for the disabled worker by recruiting and train a new worker to fill the vacant position. Thus, every injury is a cost to the organization [34] and cost affects profitability. In entirely, the cost of occupational injuries and accident on organization, workers and the society at large must not be underestimated.

Accidents and injuries alone cost the global economy 4% of total GDP ([35]); hence, there is a need for OHS to be given the needed attention. According to Concha-Barrientos et al. [36], 3.5 years of healthy life for every 1,000 workers has been lost globally through occupational injuries. Injuries remain a significant source of mortality, disability, and economic losses in the USA. The failure of management to provide safety equipment and train employees to practice health and safety at the work may lead to a rise in occupational accidents and injuries. Frequent injuries at the workplace affect employees' integrity and reduce productivity [15]. Thus, work-related injuries and productivity are inversely related.

The Canadian government spends an estimated amount of $19billion annually on occupational injuries and illness alone [37]. In the year 2000, an annual estimation of occupational accidents in 15 European Union Member States costs the economy an estimated amount of €55 billion [35]. Occupational injuries among organizations remain a public concern [38]. In China, occupational injuries remain the number one cause of death [5]. Nigeria, Sudan, Zambia, and most countries in Sub-Saharan Africa are noted to record a high rate of fatal accidents and injuries [3].

In all these, the study of Haroun et al. [27] explained that a company's investment into health and safety issues like the implementation of new safety procedures and regular provision of relevant safety apparatus significantly reduces accident and injury occurrence. An organization that provides safety equipment and educates its employees on its usage is likely to record lower work-related injuries. As organizations improve their working environment, accidents and injuries are reduced [28]. It is expected that safety program implementation and annual audits will significantly reduce work-related injuries as it remains the appropriate framework for controlling accidents [39]. Abad et al. [16] as well found occupational health and safety management as the strategic key to reduce occupational accidents and injury cost. In Ghana, there still exist no strong legal structures to govern work safety issues. More critically, accidents and injuries among organizations remain a menace which is not properly accounted for as there exist no uniform national database to record such incidents at the workplace. Based on a recent literature, the study speculates that the application of the needed safety framework in the Ghanaian oil and gas sector will reduce workplace accidents and occupational injuries.

### 2.2. Safety Knowledge

Basically, knowledge is defined as a justified belief that enhances a person's ability to take effective action or make important decisions [40]. In highly risky industries, knowledge management has been used as effective tool to transfer and share knowledge and risk experiences among workers [41, 42]. If safety requires the control of risk and hazard exposures, then safety knowledge is the ability of workers to understand these safety controls and act accordingly. Griffin and Neal [17] defined safety knowledge as the degree of knowledge about existing safety systems procedures, guidelines, and standards in the organization.

Safety knowledge has been identified among several factors such as risk perception, depression, and stress to mediate the relationship between safety behaviours and injuries [43, 44]. The emphasis on improving safety knowledge as a mechanism to promote safety behaviours and prevent work-related injuries and accidents has been championed in previous studies [17, 45–49].

According to Pyo [50], organizations must invest rigorous interest in ascertaining the required safety knowledge and its association with the specific hazards for employees; if not, using it as a risk prevention mechanism may yield conflicting results. The understanding, dissemination, and implementation of safety knowledge provide continuous feedback, recognition, improvement, and control of work-related hazards [51, 52]. Dong et al. [53] identified the importance of safety knowledge in improving safety outcomes and hence recommended the use of knowledge maps as a knowledge management tool. A good safety management system requires managers, supervisors, workers, and all other stakeholders to understand the standards and procedures of hazard prevention. From the knowledge perspective, the lack of safety knowledge is a major predictor of workplace accidents. It can therefore be assumed that effective safety knowledge management improves safety behaviour and hence a good accident prevention tool [54]. Against this backdrop, work-related accidents and injuries can be improved if the respective employees engaged in risky task acquire the needed knowledge on the existing safety systems in the organization.

## 3. Materials and Methods

A cross-sectional survey was adopted for this study. Through a convenience sampling technique, three major government-owned oil and gas companies were selected for the study. These are the Ghana National Petroleum Company (GNPC), Ghana National Gas Company (GNGC), and the Tema Oil Refinery (TOR). The convenience sampling technique was adopted to select only government enterprises. This is because there exists some form of bureaucracy among the private oil and gas sector; hence, it is quite difficult to conduct a study of such nature. All companies selected were located in the Greater Accra region, the capital city of Ghana.

**Figure 1:**
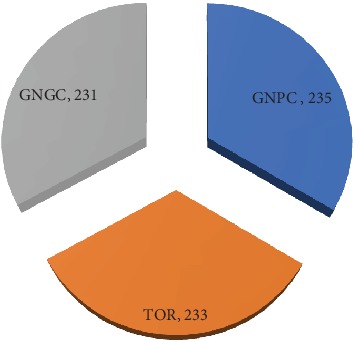
Distribution of respondents by organization.

In adhering to research ethics, letters were officially written to the three major companies to seek permission for data collection from participants. Respondents were also briefed about the objectives of the study before soliciting data. Responses to the questionnaire were willingly and voluntarily given without any duress or oppression from the organization or researchers. The sample respondents consist of workers who are directly involved in critical safety task and considered to be highly exposed to a hazardous work environment. The respondents' groups purposively consist of engineers, crane operators, maintenance personnel's, machine operators, and labourers. In all, 750 questionnaires were distributed in 3 months; however, the analysis was based on the responses of 699 oil and gas workers. Thus, 235 respondents were recruited from GNPC, 233 from TOR, and 231 from GNGC.  Figure 1 presents the respondent distribution by organization.

### 3.1. Definition of Terms

Workplace accident is basically defined as a discrete occurrence in the course of work leading to the occupational injuries [55]. In this definition lies an accident occurring while engaged in your work responsibilities or carrying out any business at work which is instructed by the employer.

Most cases of occupational injuries occur through physical, biological, chemical, or psychosocial hazards in the work environment. Such work-related hazards that can lead to injuries include noise, temperature, insect or animal bites, aerosols, blood-borne pathogens, hazardous chemicals, radiation, and occupational burnout [56]. Basically, occupational injury in this paper is defined as any form of injury or illness that is suffered by workers as related to their specific occupational demands or job requirements. In the oil and gas sector, most of the occupational injuries and accidents are expected to be resulting from fire, explosions, slips, falls, and exposure to hazardous chemicals.

Safety knowledge is defined as employees' degree of knowledge about existing safety system procedures, guidelines, and standards in the organization [17] while Occupational Health and Safety Management Frameworks (OHSMF) refer to the promotion and implementation of safety programs, procedures, and systems which are intended to eliminate or minimize the probability of risk and hazardous exposures at the workplace [57].

### 3.2. Measurement of Variables

Based on several literature reviews, the measurement of Occupation Health and Safety Management Safety Frameworks (OHSMF) was adopted from the study of Cox and Cheyne [58] and Choudhry et al. [59]. The measurement of these scales focused on assessing safety systems that will eliminate work-related risk and accidents among construction, oil, and gas workers. Five significant areas of occupational health and safety management with a test of Cronbach's reliability above 0.8 were used.

Safety knowledge was measured using a 6-item questionnaire assessing employees' degree of knowledge about existing safety frameworks adopted from the study of Griffin and Neal [17] with (*a* = .73) while the construct of occupational injuries and accidents was adopted from the study of Barling et al. [60] who also used these constructs to measure the influence of safety climate on work-related injuries.

The questionnaire was slightly modified in order to fit the measurement scale of the variables. Variable items for OHSMF were measured on a 5-point Likert scale. Thus, the measurement scale was ranked from 1 to 5. Each statement (e.g., “Safety officers and safety supervisors carry out safety inspections at regular intervals to detect hazards”) was coded as 1 = strongly disagree and 5 = strongly agree while injuries and accidents were ranked from very often to not very often (e.g., “Over the past 6months to 1year I have experienced some level of burns in my line of duty”) and will be coded as 1 = not very often and 5 = very often. Safety knowledge of employees was also measured with statements like “I understand the health and safety regulations relating to my work” and was coded as 1 = strongly disagree and 5 = strongly agree.

## 4. Results

The study used Pearson's product-moment correlation coefficient, standard multiple regression, and bootstrapping mediation method as the main method for data analysis. The presentation of the results began with the respondents' demography, as stated in Table 1.

### 4.1. Factor Loading and Reliability Test

In order to ensure that the data collected for the study are free from the violations of basic assumptions such as normality, linearity, multicollinearity, and homoscedasticity, a preliminary investigation was initially conducted.

The study adopted a convergent validity and discriminant method to validate the measurement variables. Composite reliability was utilized to test the internal consistency of the variables. Composite reliability was suitable for measuring the variable reliability because it eliminates most of the shortcomings of Cronbach's alpha (CA) [61, 62]. For the variables to be reliable, Fornell and Bookstein [63] proposed that the indicators must be significant at 0.05 and a factor loading above 0.7. Also, the average variance extracted (AVE) value must also be above 0.05.  Table 2 presents the factor loadings and reliabilities of the variables.

The factor loadings and the reliabilities of the measurement variables as indicated in Table 1 show that all the loadings are above the proposed acceptable factor loading threshold of 0.7. The lowest factor load recorded was 0.76 while the highest factor load recorded was 0.933. The average variance extracted (AVE) value also recorded a minimum of 0.712 and a maximum of 0.835, an indication that the AVE values are above the proposed AVE threshold. The findings from the table indicate that the variables for the study are highly reliable and fit for the study.

The means recorded for the constructs measuring OHSMFS does not seem encouraging. The table shows a higher means for employee priorities and need for safety (EPS) as compared to the recorded means for organizational hazards (OH); safety training (ST); safety policies, procedures and standards (SPS); and plant and equipment's/personal protection equipment (PPE). This shows that employees' value and expectations for work safety override the existing safety mechanisms or systems in the organizations.

### 4.2. Correlation and Multiple Regression Results

The correlation results were presented for the individual organizations and later combined to find the correlation results for the industry. Although not the main objective, the study seeks to find out which among the three organizations recorded the highest correlation between OHSMF and workplace injuries and accidents.

The correlation results for each organization (i.e., TOR, GNPC, and GNGC) are presented in Tables 3, 4, 5, and 6. Results gathered from TOR revealed a moderately stronger and significantly negative relationship between OHSMF and work-related injuries and accidents. Thus, the correlation between OHSMF and occupational injuries was recorded (*r* = ‐0.59, *p* < 0.05) while that between OHSMF and work place accidents was indicated (*r* = ‐0.62, *p* < 0.05). The correlation between safety knowledge and OHSMF was recorded (*r* = 0.41, *p* < 0.05) and that between safety knowledge and workplace accidents was indicated (*r* = ‐0.53, *p* < 0.05) while the correlation (*r* = ‐0.51, *p* < 0.05) between safety knowledge and occupational injuries was recorded.

The results also for GNPC recorded a significant and moderately stronger negative relationship (*r* = ‐0.57, *p* < 0.05) between OHSMF and work-related injuries. The same significant and moderately stronger negative relationship was recorded between OHSMF and workplace accidents (*r* = ‐0.63, *p* < 0.05). The correlation between safety knowledge and OHSMF was indicated (*r* = 0.39, *p* < 0.05) and that between safety knowledge and workplace accidents was indicated (*r* = ‐0.49, *p* < 0.05) while the correlation (*r* = ‐0.45, *p* < 0.05) between safety knowledge and occupational injuries was recorded.

Lastly, the correlation results of GNGC also showed a moderately strong negative relationship (*r* = 0.59, *p* < 0.05) between OHSMF and workplace accidents. The relationship between OHSMF and work-related injuries for GNGC similarly indicated a negatively strong relationship (*r* = ‐0.50, *p* < 0.05). More so, the correlation (*r* = 0.31, *p* < 0.05) between safety knowledge and OHSMF was recorded and also that (*r* = ‐0.41*p* < 0.05) between safety knowledge and workplace accidents and that (*r* = −0.37*p* < 0.05) between safety knowledge and occupational injuries.

As much as the correlation results for all the three companies recorded a moderately negative strong and significant relationship between OHSMF and work-related accidents, TOR recorded the highest correlation. The correlation between safety knowledge and OHSMF in all three organizations recorded a positively significant weak relationship, an indication that safety knowledge on safety systems among these organizations may be quite low and likely to account for the significant negative correlation recorded between OHSMF and workplace accidents and injuries.

The overall correlation for the three companies combined also revealed a strong negative and significant relationship between OHSMF systems and workplace accidents and work-related injuries (*r* = ‐0.62, *p* < 0.05; *r* = ‐0.55, *p* > 0.05) respectively. Safety knowledge recorded a weak but positive significant correlation of 0.341 with OHSMF against a significant negative correlation of -0.38 and -0.34 with workplace accident and work-related injuries, respectively.

The standard multiple regression was further adopted to predict and analyse the contribution of OHSMF on workplace accidents and work-related injuries. The results of the standard multiple regressions as shown in Table 7 revealed that OHSMF significantly contribute to both work-related injuries and workplace accidents among all three organizations in the Ghanaian oil and gas sector.

Thus, FWPA = 69.941 and FWRI = 56.788 (*p* < 0.01) were recorded for the TOR while FWPA = 68.633 and FWRI = 56.311 were recorded for GNPC. Regarding GNGC, the results reveal FWPA = 67.099 and FWRI = 55.987 (*p* < 0.01). The overall results for all the three oil and gas companies combined showed FWPA = 68.558 and FWRI = 56.362 (*p* < 0.01).

Moreover, as indicated by *R*^2^ which represents the coefficient of determination in Table 7, it can be interpreted that, OHSMF determines 52.1% (*R*^2^ = 0.521) of the variation in workplace accidents and 48.2% (*R*^2^ = 0.521) of the variation in work-related injuries in TOR. With regards to GNPC, OHSMF explained 51.1% (*R*^2^ = 0.511) and 42.3% (*R*^2^ = 0.423) of the variation in workplace accidents and work-related injuries, respectively. For GNPC, OHSMF accounted for 49.8% (*R*^2^ = 0.498) and 41.1% (*R*^2^ = 0.411), respectively, in the variation for workplace accidents and work-related injuries.

The overall results for all three companies combined revealed that OHSMF accounted for 50.1% (*R*^2^ = 0.501) for the variation in workplace accidents and 40.7% (*R*^2^ = 0.521) for the variation in work-related injuries.

### 4.3. Mediating Analysis

In order to predict the mediation role of safety knowledge in the relationship between occupational safety management framework and workplace accidents and injuries, the bootstrapping procedure was adopted. Unlike order methods of testing mediation, bootstrapping was adopted because this method generates an empirical representation of the sampling distribution of the indirect effect by treating the obtained sample of size “*n*” as a representation of the population [64]. The procedure suggested by Baron and Kenny [65] was adopted for the estimation procedure as presented in Tables 8 and 9.

The results presented in Table 8 indicated a significant total effect of -0.6899. This basically denotes the total effect of Occupational Safety Management Frameworks (OHSMF) on work-related injuries (WRI) without the mediation of safety knowledge (SK). The direct and indirect effects of SK on the causal effect of OHSMF on WRI were -0.5692 and -0.1207, respectively. The VAF value indicates that 35.18% of the total effect of OHSMF on WRI is explained by the indirect effect of SK. This suggests that the effect of OHSMF on WRI is partially mediated by SK (0.20 < VAF < 0.80). These findings support the research assumptions that anticipated safety knowledge to significantly mediate the relationship between OHSMF and WRI.

In relation to the mediation role of SK in the relationship between OHSMF and WPA, the results presented in Table 9 indicated a significant total effect of -0.7662. The direct effect of SK on OHSMF and WPA was -0.6290 while the indirect relationship recorded was -0.1372. The VAF value also indicated that 39.09% of the total effect of OHSMF on WRI is explained by the indirect effect of SK. This suggests that the effect of OHSMF on WPA is partially mediated by SK (0.20 < VAF < 0.80). These findings also support the assertion of the research objective. Thus, safety knowledge mediates the relationship between OHSMF and WPA in the Ghanaian oil and gas sector.

## 5. Discussion

As mentioned earlier, the global oil and gas industry remains one of the riskiest sectors yet very productive. The economic contribution of the oil and gas sector towards the economic development of Ghana is partly driven by employees working in this sector; hence, frequent accidents and work-related injuries are not only an organizational issue but also a national concern. Frequent work-related accidents and injuries do not only slow down work but also destroy properties which may increase replacement cost. This study therefore seeks to understand the current state of Occupational Health and Safety Management Frameworks (OHSMF) and workplace accidents and injuries. The study further used the bootstrapping method to test the mediating role of safety knowledge in the relationship between OHSMF and workplace accidents and injuries among oil and gas workers.

The study adopted a convergent validity and discriminant method to validate the measurement variables. Composite reliability was used to test the internal consistency of the variables. All factor loadings and the reliabilities of the measurement variables were above 0.7. The average variance extracted (AVE) value also recorded a minimum of 0.712 and a maximum of 0.835, an indication that the variables for the study are highly reliable and fit for the study.

The regression and correlation analysis clearly indicated a significant relationship between OHSMF and workplace accidents and injuries. Due to several health and safety policy issues or challenges chronicled in previous studies in Ghana, these findings were somehow expected; however, such a negatively strong significant relationship between OHSMF and WPA/WRI was least anticipated in such a tremendous industry. The result is an indication that the existing OHSMF in the Ghanaian oil and gas sector are either ineffective or inadequate to meet accident control measures; hence, the high rate of accident and injury was recorded. This is supported by the study of Haroun et al. [27] who posit that an organization's interest in safety and health, safety training, and safety equipment' significantly predicts work-related injuries. As organizations improve their safety systems, accidents and injuries are reduced [39]. Based on these results, specifically the findings of the regression analysis, it can be concluded that OHSMF significantly predict workplace accidents and work-related injuries in the Ghanaian oil and gas sector.

Although a weak relationship was established between OHSMF and SK, it was significant. The regression results also suggest a significant influence of OHSMF on safety knowledge. Considering the significant weak relationship between OHSMF and safety knowledge, one may argue that the mere existence of a well-established safety systems does not necessarily mean employees have the relevant safety knowledge to comply with safety rules, standards, and guidelines. This can also mean that the safety systems or programs that exist in this industry are not well implemented, established, or structured to meet the required standard and hence the resultant effect of weak correlation. As indicated by Toseafa et al. [31], workers in the Ghanaian oil and gas industry are reported to be highly exposed to hazardous chemicals, yet health and safety equipment and protective wears were inadequate for employees. The absence of safety systems distracts employees' psychological well-being and ability to perform a specific task [28]. Constructively, the reflection of this weak relationship between OHSMF and safety knowledge is likely to be a determinant of the significant negative relationship recorded between safety knowledge and workplace accidents and work-related injuries. Thus, when safety knowledge among employees is low, accident frequency is expected to increase. This supports the findings of Lette et al. [66] which revealed that the failure of organizations to provide safety equipment and educate employees on its usage will likely lead to high-rate work-related injuries. As much as management is the first to be blamed for the failure to improve job safety, employees as well have a major role to play in ensuring personal safety by abiding existing safety policies in the organization.

According to Osabutey et al. [30] apathy towards abiding to safety rules by employees remains a major concern in safety administration. Employee's safety behaviours and intentions to adhere to safety frameworks highly depend on the interest and the value the organization places on achieving good safety outcomes [67]. This assumption is also supported by Heinrich domino theory which asserts 88% of all accidents to be influenced by unsafe working habits among employees. It is likely that the failure of employees to abide existing safety policies in their respective organizations also contributed to these findings and hence the importance of safety knowledge. It is therefore expected that, if employees understand safety issues quite well, they will abide by all safety rules, standards, and procedures.

As one of the main objectives of the study, the findings showed that safety knowledge significantly mediates the relationship between OHSMF and workplace accidents just as it significantly mediates the relationship between OHSMF and work-related injuries. This supports the assumption and findings of Griffin and Neal [17] which identified safety knowledge as a determinant of safety compliance and participation in relation to organizational safety climate. These findings also confirm the findings of Murphy [47], Cole [45], Zhang et al. [49], and Toh et al. [48] who all argued on the positive influence of safety knowledge as an accident and injury prevention control tool. As Zhang and Fang [52] opined, the understanding, dissemination, and implementation of safety knowledge among workers improve the control of work-related hazards. Another significant revelation of this study also is the significant negative correlation recorded between safety training and workplace accidents and safety training and work-related injuries. This finding signals that safety training must be given the necessary attention to improve safety knowledge.

It is important to reiterate that serious catastrophes like an explosion or fire outbreak in the oil and gas sector can put the company out of business for a couple of days, months, or even years. Among most workers, such catastrophes that cause frequent injuries and accidents restrict their operational activities, whereas in some cases, these may lead to job change [67]. This is why it is important for organizations to consider safety knowledge as a key initiative through either safety training or frequent safety orientation. Safety knowledge must also be viewed and pursued as an accident prevention mechanism which must be incorporated as part of organizational culture along with the provision of effective occupational safety management systems. This is expected to produce an excellent safety performance that will subsequently reduce the frequency of workplace accidents and injuries. As Lette et al. [66] posit, an effective health and safety system is a major strategic initiative and the best decision to assist organizations in reducing cost of losing employees in the long run. This therefore calls for industry players to invest much interest in improving employees' job safety.

## 6. Conclusions

Employees' health and safety at the workplace are both psychological and emotional concern to the worker as it is directly linked to the quality of life. The findings of the study indicated that workplace accidents and work-related injuries are quite high. Likewise, the existing OHSMF may not be enough or inadequate to meet the safety needs of workers hence resulting in the negative relationship between OHSMF and WPA/WRI. Safety knowledge as well is an important factor that determines the causal relationship between OHSMF and WPA/WRI. The findings of the study call for immediate intervention from industry players in the Ghanaian oil and gas sector as well as the government or policy makers to put in all the necessary support that will improve job safety and employees' quality of life.

### 6.1. Limitations

The issue of workplace accidents and injuries in such a hazardous industry is expected to be quite complex hence may need other qualitative measures to understand the practical nature of the subject. Again, the findings will be more factual if a database on the records of accidents and injuries was considered in the analysis. Unfortunately, such a database may exist in theory but practically cumbersome to access.

The study is subjected to limitations such as social desirable and modal responses due to the self-report measures adopted for data collection. Some variable items were slightly modified to fit the intent of what was being measured; hence, the analysis is prone to common method bias. Future studies should consider investigating other data methods such as behavioural observation methods. As much as the study is a cross-sectional design, it is not enough to justify the satisfaction of the results revealed by the study. Other mediating factors such as skills and motivation, work pressure, and emotional exhaustion can be considered to strengthen the claims of the current results.

Despite these limitations, the study results are expected to be relevant to the development and improvement of safety policies, procedures, and standards that can control hazardous exposures at work and enhance the quality of life among workers. The study is significant not only to the Ghanaian oil and gas industry but also to highly risky organizations that seek to improve workers' quality of life through occupational health and safety.

## Figures and Tables

**Table 1:** Demographics of respondents.

Variable	Characteristics	Frequency	Percentage
Gender	Male	517	73.96
	Female	182	26.03

Age	20-30 years	199	28.46
	31-40 years	312	44.63
	41-50 years	151	21.60
	51 years and above	37	5.29

Education	NVTI/technical/SHS certificate	99	14.16
	HND/degree	405	57.93
	Post graduate	91	13.01
	Professional certification	85	12.16
	Other qualification	19	2.71

Working experience	1-5years	189	27.03
	6-10 years	302	43.20
	11-15years	112	16.02
	16-20years	55	7.86
	21 years and above	41	5.86

Managerial	Lower level	287	41.06
	Middle level	302	43.20
	Senior staff	110	15.73

Type of work	Engineers	88	12.58
	Maintenance personnel	211	30.18
	Labourers	127	18.16
	Crane operators	99	14.16
	Machine operators	174	24.89

**Table 2:** Factor loadings and reliabilities.

Measurement variable	Factor loadings	Means	SD	Composite reliability	AVE
*Organizational hazards (OH)*				**0.939**	**0.813**
*OH1:* safety officers and safety supervisors carry out safety inspections at regular intervals to detect hazards	0.884	1.151	0.959		
*OH2:* there exist appropriate arrangements to ensure that actions are taken because of the findings of safety inspections	0.887	2.022	0.912		
*OH3:* there exist appropriate arrangements to monitor the effectiveness and thoroughness of eliminating hazards after inspection	0.881	2.011	0.951		
*OH4:* there exist appropriate arrangements to collate and analyse the results of safety inspections after hazard elimination	0.914	1.982	0.902		
*Safety policies, procedures, and standards (SPS)*				**0.932**	**0.835**
*SPS1:* the safety policies commit the organization to full compliance with all relevant health and safety legislation	0.901	2.481	0.928		
*SPS2:* the policy set targets for health and safety performance including a commitment to progressive improvement	0.902	2.582	0.912		
*SPS3:* safety policies and procedures are explained to new employees as part of their training and orientation before entry to and work on-site	0.904	2.542	0.943		
*SPS4:* there exist effective arrangements for reviewing the health and safety policy at least once a year	0.911	1.612	0.911		
*Plant and equipment's/personal protection equipment (PPE)*				**0.935**	**0.765**
*PPE1:* all plants and equipment used on site suitable for the job and are their users properly trained	0.879	1.523	0.905		
*PPE2:* safety policies exist on the training of plant operators	0.885	2.471	0.947		
*PPE3:* there exist procedures to ensure the proper use of PPE as well as training and instruction	0.790	2.412	0.968		
*PPE4:* there is a sufficient stock of carefully selected and appropriate PPE at all times	0.760	1.373	0.806		
*Safety training (ST)*				**0.936**	**0.769**
*ST1:* my company gives comprehensive training to the employees in workplace health and safety issues	0.877	2.451	1.011		
*ST2:* newly recruits are trained adequately to learn safety rules and procedures	0.877	2.512	0.983		
*ST3:* safety training given to me is adequate to enable me to assess hazards in workplace	0.873	2.482	0.967		
*ST4:* safety issues are given high priority in training programs	0.872	2.424	0.973		
*Employee priorities and need for safety (EPS)*				**0.947**	**0.805**
*EPS1:* a safe place to work has a lot of personal meaning to me	0.887	4.173	1.069		
*EPS2:* it is important that there is a continuing emphasis on safety	0.859	4.071	1.053		
*EPS3:* I understand the safety rules for my job	0.884	3.471	1.058		
*EPS4:* safety is the number one priority in my mind when completing a job	0.923	4.394	1.078		
*Safety knowledge (SK)*				**0.812**	**0.642**
*SK1:* I know how to perform my job in a safe manner	0.812	3.022	0.561		
*SK2:* I know how to use safety equipment and standard work procedures	0.799	3.192	0.652		
*SK3:* I know how to maintain or improve workplace health and safety	0.809	2.772	0.622		
*SK4:* I know how to reduce the risk of accidents and incidents in the workplace	0.823	3.051	0.712		
*SK5:* I know what are the hazards associated with my jobs and the necessary precautions to be taken while doing my job.	0.877	3.272	0.577		
*SK6:* I do not know what to do and whom to report if a potential hazard is noticed in my workplace.	0.811	3.112	0.601		
*Workplace accidents (WA)*				**0.822**	**0.721**
*WPA1:* over the past 6 months to 1 year I have been struck against something fixed or stationary	0.891	3.012	0.919		
*WPA2:* over the past 6 months to 1 year I have overextended myself lifting or moving things	0.901	3.96	0.812		
*WPA3:* over the past 6 months to 1 year I have been exposed to chemicals such as gases and fumes.	0.933	4.113	0.911		
*WPA4:* over the past 6 months to 1 year I have fell off of something (e.g., a ladder, shelf, etc.)	0.921	3.735	0.922		
*WPA5:* over the past 6 months to 1 year I have been trapped by something collapsing, caving in or overturning	0.891	2.771	0.933		
*WPA6:* over the past 6 months to 1 year I have slipped and fell whiles going about my work duties	0.877	3.113	1.721		
*Work-related injuries (WRI)*				**0. 893**	**0.712**
*WRI1:* over the past 6 months to 1 year I have experienced some level of burns in my line of duty	0.908	2.112	0.912		
*WRI2:* over the past 6 months to 1 year I have experienced strains or sprains	0.913	2.785	0.978		
*WRI3:* over the past 6 months to 1 year I have had cuts or lacerations	0.792	3.012	0.982		
*WRI4:* over the past 6 months to 1 year I have experienced serious muscle or back pain	0.897	4.434	0.943		
*WRI5:* over the past 6 months to 1 year I have had bruises or contusions	0.912	3.014	0.957		
*WRI6:* over the past 6 months to 1 year I have experienced a dislocation of joint due to my line of work	0.813	3.182	1.089		

**Table 3:** Correlation results for Tema Oil Refinery (TOR).

Variables	1	2	3	4	5	6	7	8	9	10	11	12	13	14
(1) Gender	—													
(2) Age	.236													
(3) Education	.458	.692												
(4) Work experience	.558	.525	.314											
(5) Managerial level	.414	.336	.281	.582										
(6) Organizational hazards (OH)	.571	.513	.225	.458	.447									
(7) Safety policies, procedures, and standards (SPS)	.436	.292	.258	.381	.369	.714								
(8) Plant and Equipment's/personal protection equipment (PPE)	.271	.446	.171	.425	.358	.663	.614							
(9) Safety training (ST)	.481	.413	.192	.414	.371	.614	.592	.571						
(10) Employee priorities and need for safety (EPS)	.669	.569	.492	.614	.582	-.458	-.414	-.337	-.292					
(11) Occupational safety management framework (OHSMF)	.514	.471	.336	.371	.214	.669	.614	.558	.592	-.447				
(12) Safety knowledge (SK)	.114	.225	.392	.271	.247	.414	.393	.414	.271	-.293	**.413**			
(13) Workplace accidents (WPA)	.158	.181	.225	.292	.271	-.514	-.559	-.447	-.614	-.437	**-.628**	**-.535**		
(14) Work-related injuries (WRI)	-.514	-.124	.192	.236	.214	-.492	-.525	-.419	-.593	-.392	**-.592**	**-.512**	**.893**	—

Correlations above 0.3 and -0.3 were all significant at ^∗^*p* < 0.05 (interpretation of results was based on 2 decimals).

**Table 4:** Correlation results for Ghana National Petroleum Company (GNPC).

Variables	1	2	3	4	5	6	7	8	9	10	11	12	13	14
(1) Gender	—													
(2) Age	.216													
(3) Education	.437	.675												
(4) Work experience	.535	.542	.331											
(5) Managerial level	.434	.315	.263	.561										
(6) Organizational hazards (OH)	.553	.534	.194	.431	.423									
(7) Safety policies, procedures, and standards (SPS)	.414	.279	.237	.362	.341	.732								
(8) Plant and equipment's/personal protection equipment (PPE)	.254	.423	.153	.403	.338	.634	.595							
(9) Safety training (ST)	.465	.434	.179	.433	.359	.593	.573	.557						
(10) Employee priorities and need for safety (EPS)	.648	.545	.474	.634	.567	-.431	-.431	-.319	-.279					
(11) Occupational safety management framework (OHSMF)	.534	.457	.313	.354	.235	.644	.638	.532	.577	-.412				
(12) Safety knowledge (SK)	.135	.176	.313	.177	.219	.391	.346	.364	.215	-.274	**.391**			
(13) Workplace accidents (WPA)	-.534	.166	.252	.277	.247	-.544	-.574	-.416	-.633	.356	**-.633**	**-.492**		
(14) Work-related injuries (WRI)	-.535	-.154	.174	.259	.235	-.472	-.542	-.438	-.561	.318	**-.571**	**-.454**	**.772**	—

Correlations above 0.3 and -0.3 were all significant at ^∗^*p* < 0.05 (interpretation of results was based on 2 decimals).

**Table 5:** Correlation results for Ghana National Gas Company (GNGC).

Variables	1	2	3	4	5	6	7	8	9	10	11	12	13	14
(1) Gender	—													
(2) Age	.122													
(3) Education	.324	.562												
(4) Work experience	.413	.424	.222											
(5) Managerial level	.435	.313	.268	.442										
(6) Organizational hazards (OH)	.435	.413	.091	.313	.312									
(7) Safety policies, procedures, and standards (SPS)	.291	.113	.179	.235	.279	.613								
(8) Plant and equipment's/personal protection equipment (PPE)	.335	.324	.157	.402	.235	.535	.513							
(9) Safety training (ST)	.368	.312	.279	.213	.257	.491	.479	.446						
(10) Employee priorities and need for safety (EPS)	.546	.446	.357	.524	.435	-.313	-.291	-.257	-.279					
(11) Occupational safety management framework (OHSMF)	.435	.357	.224	.268	.213	.513	.535	.532	.457	-.379				
(12) Safety knowledge (SK)	.135	.179	.313	.179	.213	.391	.346	.368	.235	-.279	**.313**			
(13) Workplace accidents (WPA)	-.413	.112	.168	.192	.191	-.412	-.435	-.224	-.512	-.413	**-.591**	**-.413**		
(14) Work-related injuries (WRI)	-.413	-.135	.113	.257	.135	-.324	-.413	-.313	-.435	-.413	**-.502**	**-.371**	**.724**	**—**

Correlations above 0.3 and -0.3 were all significant at ^∗^*p* < 0.05 (interpretation of results was based on 2 decimals).

**Table 6:** Combined correlation for the oil and gas industry.

Overall (TOR, GNPC, GNGC)	WPA	WRI	SK
Workplace accidents	1		
Work-related injuries	.797^∗∗^	1	
Safety knowledge	-.389^∗∗^	-.342^∗∗^	1
Occupational Health and Safety Management Frameworks(OHSMF)	-.627^∗∗^	-.553^∗^	.341^∗^

^∗∗^ *p* < 0.01 and ^∗^*p* < 0.05. WPA: workplace accidents; WRI: work-related injuries; SK: safety knowledge.

**Table 7:** Standard multiple regression results.

TOR	*β*	*R* ^2^	*F*	*T*
OHSMF and WPA	-0.657	0.521	65.941^∗^	-7.681^∗^
OHSMF and WRI	-0.591	0.482	56.788^∗^	-5.201^∗^
OHSMF and SK	0.411	0.344	6.022^∗^	2.807^∗^
WPA and SK	-0.537	0.471	41.301^∗^	-3.877^∗^
WRI and SK	-0.517	0.429	39.009^∗^	-2.996^∗^
WPA and WRI	0.893	0.881	69.003^∗^	5.099^∗^
GNPC				
OHSMF and WPA	-0.632	0.511	63.633^∗^	-7.301^∗^
OHSMF and WRI	-0.517	0.423	56.311^∗^	-6.211^∗^
OHSMF and SK	0.390	0.355	5.212^∗^	2.611^∗^
WPA and SK	-0.491	0.427	32.001^∗^	-3.117^∗^
WRI and SK	-0.452	0.399	27.333^∗^	-3.001^∗^
WPA and WRI	0.772	0.654	64.911^∗^	4.749^∗^
GNGC				
OHSMF and WPA	-0.599	0.498	57.099^∗^	-6.993^∗^
OHSMF and WRI	-0.503	0.411	55.987^∗^	-5.721^∗^
OHSMF and SK	0.313	0.277	3.180^∗^	2.337^∗^
WPA and SK	-0.416	0.361	22.777^∗^	-3.111^∗^
WRI and SK	-0.371	0.324	17.219^∗^	-2.317^∗^
WPA and WRI	0.722	0.666	55.007^∗^	4.028^∗^
OVERALL (TOR, GNPC, GNGC)				
OHSMF and WPA	**-0.629**	0.501	61.558^∗^	-6.711^∗^
OHSMF and WRI	**-0.549**	0.407	56.362^∗^	-5.292^∗^
OHSMF and SK	**0.351**	0.311	4.312^∗^	3.711^∗^
WPA and SK	**-0.391**	0.350	26.221^∗^	-4.511^∗^
WRI and SK	**-0.344**	0.296	19.231^∗^	-3.552^∗^
WPA and WRI	**0.796**	0.633	68.917^∗^	5.002^∗^

^∗^ *p* < 0.01. WPA: workplace accidents; WRI: work-related injuries; OHSMF: Occupational Safety Management Framework; SK: safety knowledge.

**Table 8:** Results of the mediating analysis (OHSMF-SK-WRI).

	Estimate	95% CI lower	95% CI upper	*p* value
Direct	-0.5692	-0.5966	0.6392	0.0009
Indirect	-0.1207	-0.1872	0.3599	0.0241
Total effect	-0.6899	-0.7673	0.6895	0.0123
Prop. mediated	0.6000	0.6365	0.9921	0.0005
VAF	35.18			

Note: WPA = workplace accidents; WRI = work-related injuries; OHSMF = Occupational Safety Management Frameworks; SK = safety knowledge; VAF = variation accounted f4or.

**Table 9:** Results of the mediating analysis (OHSMF-SK-WPA).

	Estimate	95% CI lower	95% CI upper	*p* value
Direct	-0.6290	-0.6366	0.6392	0.0007
Indirect	-0.1372	-0.1872	0.3599	0.0231
Total effect	-0.7662	-0.9673	0.6895	0.0113
Prop. mediated	0.6000	0.7365	1.9921	0.0001
VAF	39.09			

Note: WPA = workplace accidents; WRI = work-related injuries; OHSMF = Occupational Safety Management Frameworks; SK = safety knowledge; VAF = variation accounted for.

## Data Availability

The data used to support the findings of this study are restricted and confidential. Researchers who meet the criteria for access to confidential data can privately contact the corresponding author.
